# 1′-(1,3-Diphenyl-1*H*-pyrazol-4-yl)-1′′-methyl-2′,3′,5′,6′,7′,7a’-octa­hydro-1′*H*-dispiro­[1-benzopyran-3,2′-pyrrolizine-3′,3′′-indoline]-2′′,4-dione

**DOI:** 10.1107/S1600536813002043

**Published:** 2013-02-02

**Authors:** G. Jagadeesan, K. Sethusankar, D. Kathirvelan, J. Haribabu, B. S. R. Reddy

**Affiliations:** a Department of Physics, Meenakshi College of Engineering, West K.K. Nagar, Chennai 600 078, India; b Department of Physics, RKM Vivekananda College (Autonomous), Chennai 600 004, India; c Industrial Chemistry Lab, Central Leather Research Institute, Adyar, Chennai 600 020, India

## Abstract

In the title compound C_38_H_32_N_4_O_3_, one pyrrolidine ring adopts an envelope conformation with the N atom as the flap while other pyrrolidine ring adopts an twisted conformation. The pyrrolizine ring forms dihedral angles of 79.24 (5) and 77.57 (5)° with the chromene and indole rings, respectively. The carbonyl O atoms deviate from the least-square planes through the chromene and indole rings by 0.0113 (12) and 0.0247 (12) Å, respectively. In the crystal, non-classical C—H⋯O inter­actions link the mol­ecules, generating an *C*(9) chain along the *b*-axis direction.

## Related literature

For the biological activity of pyrazole derivatives, see: Mahajan *et al.* (1991 ▶); Baraldi *et al.* (1998 ▶); Katayama & Oshiyama (1997 ▶); Chen & Li (1998 ▶). For a related structure, see: Fun *et al.* (2011 ▶). For puckering parameters, see: Cremer & Pople (1975 ▶).
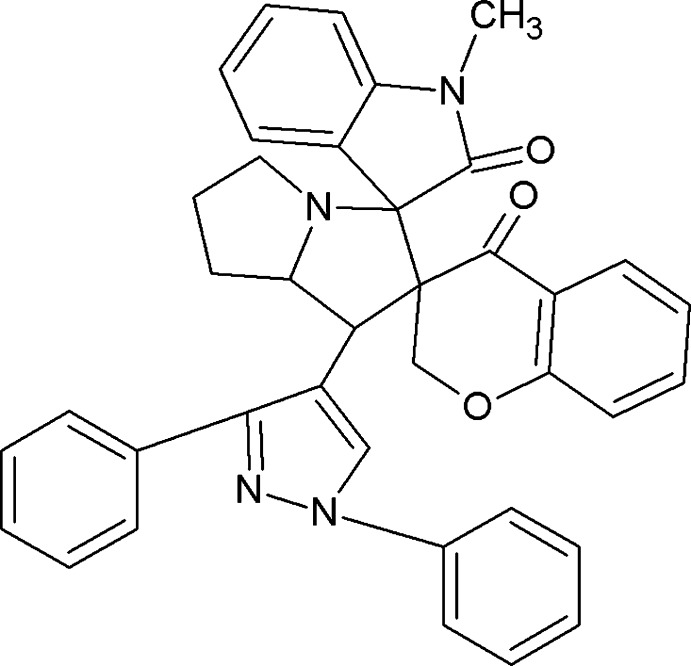


## Experimental

### 

#### Crystal data

C_38_H_32_N_4_O_3_
*M* *_r_* = 592.68
Triclinic, 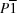

*a* = 10.8240 (3) Å
*b* = 10.8382 (3) Å
*c* = 13.9127 (4) Å
α = 70.290 (1)°
β = 88.946 (2)°
γ = 73.578 (1)°
*V* = 1468.47 (7) Å^3^
*Z* = 2
Mo *K*α radiation
μ = 0.09 mm^−1^
*T* = 295 K
0.30 × 0.25 × 0.20 mm

#### Data collection

Bruker Kappa APEXII CCD diffractometer
Absorption correction: multi-scan (*SADABS*; Bruker, 2008 ▶) *T*_min_ = 0.975, *T*_max_ = 0.983
35301 measured reflections
9340 independent reflections
5865 reflections with *I* > 2σ(*I*)
*R*_int_ = 0.032

#### Refinement

*R*[*F* ^2^ > 2σ(*F* ^2^)] = 0.055
*wR*(*F* ^2^) = 0.166
*S* = 1.06
9340 reflections
406 parameters
H-atom parameters constrained
Δρ_max_ = 0.35 e Å^−3^
Δρ_min_ = −0.25 e Å^−3^

### 

Data collection: *APEX2* (Bruker, 2008 ▶); cell refinement: *SAINT* (Bruker, 2008 ▶); data reduction: *SAINT*; program(s) used to solve structure: *SHELXS97* (Sheldrick, 2008 ▶); program(s) used to refine structure: *SHELXL97*; molecular graphics: *ORTEP-3 for Windows* (Farrugia, 2012 ▶); software used to prepare material for publication: *SHELXL97* (Sheldrick, 2008 ▶) and *PLATON* (Spek, 2009 ▶).

## Supplementary Material

Crystal structure: contains datablock(s) global, I. DOI: 10.1107/S1600536813002043/rk2391sup1.cif

Structure factors: contains datablock(s) I. DOI: 10.1107/S1600536813002043/rk2391Isup2.hkl

Additional supplementary materials:  crystallographic information; 3D view; checkCIF report

## Figures and Tables

**Table 1:** Hydrogen-bond geometry (Å, °)

*D*—H⋯*A*	*D*—H	H⋯*A*	*D*⋯*A*	*D*—H⋯*A*
C34—H33⋯O3^i^	0.93	2.59	3.523 (3)	178

Symmetry code: (i) 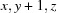.

**Fig. 1.**
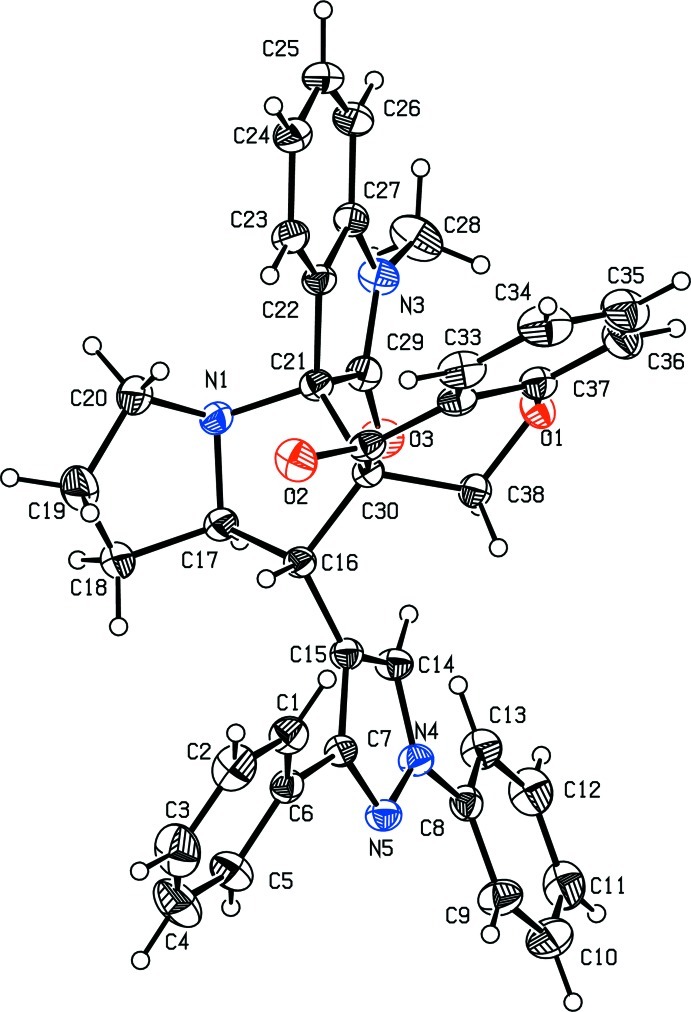
The molecular structure of the title compound with the atom numbering scheme. Displacement ellipsoids are drawn at 30% probability level. H atoms are presented as a small spheres of arbitrary radius.

**Fig. 2.**
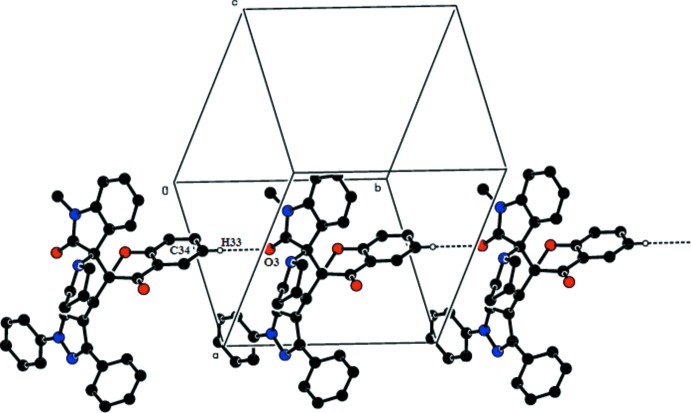
The crystal packing of the title compound viewed down a axis, dashed lines indicates C–H···O interactions. H atoms not involved in hydrogen bonds have been excluded for clarity.

## supplementary crystallographic information

### Comment

Pyrazole derivatives in general are well known nitrogen containing heterocyclic compounds and these derivatives have been the subject of enormous research due to their importance in various applications and their widespread potential biological and pharmacological activities such as antimicrobial (Mahajan *et al.*, 1991), antiviral (Baraldi *et al.*, 1998), antitumor (Katayama & Oshiyama, 1997), antifungal activities (Chen & Li, 1998).

The molecular structure of the title compound C_38_H_32_N_4_O_3_, is shown in Fig. 1. The phenyl rings (C1-C6) and (C8-C13) attached with the pyrazole ring (C7/C14/C15/N4/N5) form a dihedral angle of 52.60 (6)° between them. The pyrazole ring (C7/C14/C15/N4/N5) forms dihedral angles of 41.24 (6)° and 12.85 (5)° with the two phenyl rings (C1-C6) and (C8-C13), respectively. The pyrrolizine ring (C16-C21/C30/N1) forms dihedral angles of 79.24 (5)° and 77.57 (5)° with the chromene ring (C30-C38/O1) and indole ring (C21-C27/C29/N3), respectively. The atoms C28, O3 and O2 deviate from the l.s. planes of the indole ring (C21-C27/C29/N3) and chromene ring (C30-C38/O1) by 0.020 (2)Å, 0.0247 (12)Å and 0.0113 (12)Å, respectively. The title compound exhibits the structural similarities with the already reported related structure (Fun *et al.*, 2011).

The sum of angles around the N_1_ atom (340°) indicates *sp*^3^ hybridization. The pyrrolidine ring (C16/C17/C21/C30/N1) adopts an *envelope* conformation on N1 with puckering parameters (Cremer & Pople, 1975) of q_2_ = 0.3225 (15)Å and φ_2_= 186.4 (3)°. Also, the atom N1 deviates from the mean planes of the remaining ring atoms by -0.2027 (14)Å. The other pyrrolidine ring (C17-C20/N1) adopts a *twisted* conformation on C17 and C18 with puckering parameters of q_2_ = 0.294 (2)Å and φ_2_ = 236.2 (4)°. Also, the atoms C17 and C18 deviate from the mean planes of the remaining ring atoms by 0.1743 (17)Å and -0.179 (2)Å.

The crystal packing is stabilized by non-classical C–H···O interactions (Table 1). The C34-H33···O3^i^ interaction generates a *C*(9) chain along the *b* axis. The symmetry code: (i) *x*, *y*+1, *z*. The packing view of the compound is shown in Fig. 2.

### Experimental

A mixture of methyl isatin (1.05 mmol), sarcosine (1.1 mmol), dipolarophile (1.0 mmol) in ethanol was refluxed for 85 min and cooled to room temperature. Then the mixture was poured into crushed ice breaker and the solid formed in the mixture was filtered, dried, and recrystallized from ethanol to obtain the pure product in good yield 93%.

### Refinement

Hydrogen atoms were placed in calculated positions with C–H = 0.93-0.98Å and refined in the riding model with fixed isotropic displacement parameters: *U*_iso_(H) = 1.5*U*_eq_(C) for methyl group and *U*_iso_(H) = 1.2*U*_eq_(C) for other groups.

### Crystal data

**Table:**

C_38_H_32_N_4_O_3_	*Z* = 2
*M**_r_* = 592.68	*F*(000) = 624
Triclinic, *P*1	*D*_x_ = 1.340 Mg m^−^^3^
Hall symbol: -P 1	Mo *K*α radiation, λ = 0.71073 Å
*a* = 10.8240 (3) Å	Cell parameters from 9340 reflections
*b* = 10.8382 (3) Å	θ = 2.1–31.2°
*c* = 13.9127 (4) Å	µ = 0.09 mm^−^^1^
α = 70.290 (1)°	*T* = 295 K
β = 88.946 (2)°	Block, colourless
γ = 73.578 (1)°	0.30 × 0.25 × 0.20 mm
*V* = 1468.47 (7) Å^3^	

### Data collection

**Table:**

Bruker Kappa APEXII CCD diffractometer	9340 independent reflections
Radiation source: fine-focus sealed tube	5865 reflections with *I* > 2σ(*I*)
Graphite monochromator	*R*_int_ = 0.032
ω scans	θ_max_ = 31.2°, θ_min_ = 2.1°
Absorption correction: multi-scan (*SADABS*; Bruker, 2008)	*h* = −15→15
*T*_min_ = 0.975, *T*_max_ = 0.983	*k* = −15→15
35301 measured reflections	*l* = −20→19

### Refinement

**Table:**

Refinement on *F*^2^	Primary atom site location: structure-invariant direct methods
Least-squares matrix: full	Secondary atom site location: difference Fourier map
*R*[*F*^2^ > 2σ(*F*^2^)] = 0.055	Hydrogen site location: inferred from neighbouring sites
*wR*(*F*^2^) = 0.166	H-atom parameters constrained
*S* = 1.06	*w* = 1/[σ^2^(*F*_o_^2^) + (0.0793*P*)^2^ + 0.1729*P*] where *P* = (*F*_o_^2^ + 2*F*_c_^2^)/3
9340 reflections	(Δ/σ)_max_ < 0.001
406 parameters	Δρ_max_ = 0.35 e Å^−^^3^
0 restraints	Δρ_min_ = −0.25 e Å^−^^3^

### Special details

**Table:**

Geometry. All s.u.'s (except the s.u. in the dihedral angle between two l.s. planes) are estimated using the full covariance matrix. The cell s.u.'s are taken into account individually in the estimation of s.u.'s in distances, angles and torsion angles; correlations between s.u.'s in cell parameters are only used when they are defined by crystal symmetry. An approximate (isotropic) treatment of cell s.u.'s is used for estimating s.u.'s involving l.s. planes.
Refinement. Refinement of *F*^2^ against ALL reflections. The weighted *R*-factor *wR* and goodness of fit *S* are based on *F*^2^, conventional *R*-factors *R* are based on *F*, with *F* set to zero for negative *F*^2^. The threshold expression of *F*^2^ > σ(*F*^2^) is used only for calculating *R*-factors(gt) *etc*. and is not relevant to the choice of reflections for refinement. *R*-factors based on *F*^2^ are statistically about twice as large as those based on *F*, and *R*-factors based on ALL data will be even larger.

### Fractional atomic coordinates and isotropic or equivalent isotropic displacement parameters (Å^2^)

**Table:**

	*x*	*y*	*z*	*U*_iso_*/*U*_eq_	
C1	1.20015 (16)	0.45544 (16)	0.12448 (12)	0.0447 (4)	
H1	1.1229	0.5038	0.1421	0.054*	
C2	1.3123 (2)	0.4889 (2)	0.13459 (15)	0.0582 (5)	
H2	1.3102	0.5594	0.1589	0.070*	
C3	1.4264 (2)	0.4182 (2)	0.10881 (18)	0.0723 (6)	
H3	1.5022	0.4389	0.1174	0.087*	
C4	1.42844 (19)	0.3164 (2)	0.07008 (19)	0.0758 (6)	
H4	1.5054	0.2702	0.0507	0.091*	
C5	1.31727 (17)	0.28249 (19)	0.05992 (15)	0.0543 (4)	
H5	1.3197	0.2134	0.0338	0.065*	
C6	1.20183 (14)	0.35058 (14)	0.08829 (11)	0.0365 (3)	
C7	1.08498 (13)	0.30861 (14)	0.08134 (11)	0.0335 (3)	
C8	0.90446 (14)	0.17479 (14)	−0.03996 (11)	0.0372 (3)	
C9	0.94126 (19)	0.19600 (19)	−0.13752 (13)	0.0521 (4)	
H9	1.0018	0.2431	−0.1609	0.063*	
C10	0.8874 (2)	0.1466 (2)	−0.20098 (16)	0.0649 (5)	
H10	0.9118	0.1614	−0.2674	0.078*	
C11	0.7992 (2)	0.0766 (2)	−0.16741 (17)	0.0626 (5)	
H11	0.7643	0.0429	−0.2103	0.075*	
C12	0.76284 (19)	0.05675 (19)	−0.07089 (16)	0.0587 (5)	
H12	0.7018	0.0101	−0.0482	0.070*	
C13	0.81477 (17)	0.10454 (17)	−0.00578 (14)	0.0489 (4)	
H13	0.7896	0.0896	0.0605	0.059*	
C14	0.91574 (14)	0.23330 (15)	0.11587 (11)	0.0380 (3)	
H14	0.8460	0.2064	0.1464	0.046*	
C15	0.99309 (13)	0.28870 (14)	0.15423 (11)	0.0338 (3)	
C16	0.98987 (13)	0.30356 (13)	0.25733 (10)	0.0319 (3)	
H16	1.0618	0.3383	0.2658	0.038*	
C17	1.01160 (14)	0.16187 (14)	0.34315 (11)	0.0374 (3)	
H17	0.9760	0.1025	0.3194	0.045*	
C18	1.15159 (16)	0.08961 (18)	0.38548 (13)	0.0510 (4)	
H18A	1.1669	−0.0088	0.4150	0.061*	
H18B	1.2098	0.1090	0.3323	0.061*	
C19	1.16913 (18)	0.1481 (2)	0.46624 (16)	0.0677 (6)	
H19A	1.2281	0.0789	0.5229	0.081*	
H19B	1.2041	0.2248	0.4380	0.081*	
C20	1.03935 (16)	0.1942 (2)	0.50153 (13)	0.0508 (4)	
H20A	1.0344	0.1345	0.5705	0.061*	
H20B	1.0223	0.2870	0.5015	0.061*	
C21	0.82823 (13)	0.30387 (14)	0.38591 (10)	0.0322 (3)	
C22	0.76483 (14)	0.36324 (14)	0.46523 (11)	0.0347 (3)	
C23	0.79918 (15)	0.43927 (16)	0.51647 (11)	0.0407 (3)	
H23	0.8702	0.4719	0.4990	0.049*	
C24	0.72698 (17)	0.46666 (18)	0.59423 (12)	0.0474 (4)	
H24	0.7498	0.5180	0.6288	0.057*	
C25	0.62210 (17)	0.41859 (19)	0.62049 (13)	0.0529 (4)	
H25	0.5754	0.4368	0.6734	0.063*	
C26	0.58482 (16)	0.34373 (19)	0.56975 (13)	0.0502 (4)	
H26	0.5133	0.3119	0.5873	0.060*	
C28	0.53123 (18)	0.1847 (2)	0.43800 (18)	0.0645 (5)	
H27A	0.4777	0.2036	0.4901	0.097*	
H27B	0.5660	0.0874	0.4550	0.097*	
H27C	0.4805	0.2233	0.3733	0.097*	
C29	0.72848 (14)	0.23509 (14)	0.36510 (11)	0.0368 (3)	
C30	0.86386 (13)	0.39982 (13)	0.28047 (10)	0.0294 (3)	
C31	0.89175 (14)	0.52146 (14)	0.29848 (10)	0.0341 (3)	
C32	0.78405 (15)	0.64959 (14)	0.27149 (11)	0.0378 (3)	
C33	0.7986 (2)	0.76442 (17)	0.28913 (14)	0.0514 (4)	
H32	0.8775	0.7606	0.3172	0.062*	
C34	0.6973 (2)	0.88303 (19)	0.26547 (16)	0.0662 (6)	
H33	0.7080	0.9590	0.2773	0.079*	
C35	0.5800 (2)	0.88894 (19)	0.22412 (17)	0.0663 (5)	
H34	0.5120	0.9694	0.2080	0.080*	
C36	0.56248 (19)	0.77743 (18)	0.20650 (15)	0.0551 (4)	
H35	0.4831	0.7819	0.1789	0.066*	
C37	0.66482 (15)	0.65802 (15)	0.23050 (12)	0.0396 (3)	
C38	0.75803 (14)	0.45991 (14)	0.19302 (11)	0.0352 (3)	
H37A	0.7906	0.5106	0.1314	0.042*	
H37B	0.7363	0.3856	0.1802	0.042*	
N1	0.94466 (12)	0.18845 (12)	0.42931 (9)	0.0362 (3)	
C27	0.65651 (14)	0.31774 (15)	0.49263 (11)	0.0384 (3)	
N3	0.63590 (12)	0.24491 (13)	0.43134 (10)	0.0424 (3)	
N4	0.95794 (12)	0.22443 (12)	0.02610 (9)	0.0359 (3)	
N5	1.06305 (12)	0.27041 (12)	0.00305 (9)	0.0372 (3)	
O1	0.64346 (10)	0.54909 (10)	0.21348 (8)	0.0419 (3)	
O2	0.99610 (11)	0.51308 (12)	0.33489 (9)	0.0505 (3)	
O3	0.73360 (11)	0.17644 (12)	0.30382 (9)	0.0491 (3)	

### Atomic displacement parameters (Å^2^)

**Table:**

	*U*^11^	*U*^22^	*U*^33^	*U*^12^	*U*^13^	*U*^23^
C1	0.0454 (9)	0.0474 (8)	0.0443 (9)	−0.0211 (7)	0.0063 (7)	−0.0136 (7)
C2	0.0624 (12)	0.0633 (11)	0.0570 (11)	−0.0352 (9)	0.0015 (9)	−0.0170 (9)
C3	0.0483 (11)	0.0859 (15)	0.0876 (16)	−0.0369 (11)	−0.0011 (10)	−0.0214 (12)
C4	0.0356 (10)	0.0840 (15)	0.1056 (18)	−0.0183 (10)	0.0170 (10)	−0.0299 (13)
C5	0.0407 (9)	0.0576 (10)	0.0662 (12)	−0.0152 (8)	0.0131 (8)	−0.0233 (9)
C6	0.0354 (7)	0.0395 (7)	0.0319 (7)	−0.0149 (6)	0.0040 (6)	−0.0059 (6)
C7	0.0336 (7)	0.0340 (6)	0.0315 (7)	−0.0102 (5)	0.0043 (5)	−0.0094 (5)
C8	0.0376 (8)	0.0343 (7)	0.0388 (8)	−0.0065 (6)	−0.0005 (6)	−0.0147 (6)
C9	0.0602 (11)	0.0633 (10)	0.0443 (9)	−0.0247 (9)	0.0102 (8)	−0.0278 (8)
C10	0.0746 (14)	0.0794 (13)	0.0519 (11)	−0.0200 (11)	0.0041 (10)	−0.0388 (10)
C11	0.0603 (12)	0.0649 (11)	0.0723 (13)	−0.0111 (9)	−0.0119 (10)	−0.0410 (10)
C12	0.0560 (11)	0.0580 (10)	0.0727 (13)	−0.0235 (9)	−0.0015 (9)	−0.0300 (10)
C13	0.0513 (10)	0.0517 (9)	0.0503 (10)	−0.0212 (8)	0.0037 (8)	−0.0209 (8)
C14	0.0378 (8)	0.0451 (8)	0.0369 (8)	−0.0175 (6)	0.0097 (6)	−0.0175 (6)
C15	0.0335 (7)	0.0363 (7)	0.0324 (7)	−0.0112 (5)	0.0047 (5)	−0.0121 (5)
C16	0.0298 (7)	0.0378 (7)	0.0308 (7)	−0.0131 (5)	0.0043 (5)	−0.0125 (5)
C17	0.0396 (8)	0.0373 (7)	0.0332 (7)	−0.0105 (6)	0.0049 (6)	−0.0102 (6)
C18	0.0431 (9)	0.0510 (9)	0.0440 (9)	0.0003 (7)	0.0036 (7)	−0.0093 (7)
C19	0.0425 (10)	0.0907 (15)	0.0651 (13)	−0.0051 (10)	−0.0079 (9)	−0.0323 (11)
C20	0.0420 (9)	0.0666 (10)	0.0425 (9)	−0.0129 (8)	−0.0028 (7)	−0.0194 (8)
C21	0.0319 (7)	0.0362 (6)	0.0319 (7)	−0.0157 (5)	0.0054 (5)	−0.0115 (5)
C22	0.0335 (7)	0.0408 (7)	0.0304 (7)	−0.0140 (6)	0.0072 (5)	−0.0109 (6)
C23	0.0402 (8)	0.0508 (8)	0.0359 (8)	−0.0182 (7)	0.0062 (6)	−0.0172 (7)
C24	0.0468 (9)	0.0586 (9)	0.0398 (8)	−0.0131 (7)	0.0049 (7)	−0.0229 (7)
C25	0.0457 (9)	0.0708 (11)	0.0418 (9)	−0.0125 (8)	0.0141 (7)	−0.0235 (8)
C26	0.0375 (8)	0.0648 (10)	0.0470 (9)	−0.0184 (7)	0.0146 (7)	−0.0156 (8)
C28	0.0468 (10)	0.0686 (12)	0.0983 (16)	−0.0378 (9)	0.0225 (10)	−0.0377 (11)
C29	0.0372 (8)	0.0363 (7)	0.0389 (8)	−0.0172 (6)	0.0054 (6)	−0.0102 (6)
C30	0.0294 (6)	0.0337 (6)	0.0285 (6)	−0.0139 (5)	0.0031 (5)	−0.0110 (5)
C31	0.0369 (7)	0.0426 (7)	0.0304 (7)	−0.0215 (6)	0.0078 (6)	−0.0140 (6)
C32	0.0470 (9)	0.0370 (7)	0.0350 (7)	−0.0196 (6)	0.0121 (6)	−0.0140 (6)
C33	0.0688 (12)	0.0483 (9)	0.0525 (10)	−0.0307 (8)	0.0178 (9)	−0.0259 (8)
C34	0.0953 (17)	0.0428 (9)	0.0710 (13)	−0.0274 (10)	0.0283 (12)	−0.0281 (9)
C35	0.0771 (15)	0.0425 (9)	0.0727 (13)	−0.0060 (9)	0.0175 (11)	−0.0218 (9)
C36	0.0497 (10)	0.0483 (9)	0.0594 (11)	−0.0049 (7)	0.0085 (8)	−0.0166 (8)
C37	0.0438 (8)	0.0382 (7)	0.0377 (8)	−0.0134 (6)	0.0097 (6)	−0.0134 (6)
C38	0.0348 (7)	0.0377 (7)	0.0344 (7)	−0.0112 (6)	0.0011 (6)	−0.0135 (6)
N1	0.0357 (6)	0.0400 (6)	0.0302 (6)	−0.0121 (5)	0.0037 (5)	−0.0081 (5)
C27	0.0336 (7)	0.0423 (7)	0.0378 (8)	−0.0137 (6)	0.0059 (6)	−0.0102 (6)
N3	0.0360 (7)	0.0476 (7)	0.0512 (8)	−0.0237 (6)	0.0116 (6)	−0.0176 (6)
N4	0.0362 (6)	0.0417 (6)	0.0339 (6)	−0.0146 (5)	0.0054 (5)	−0.0159 (5)
N5	0.0361 (6)	0.0444 (6)	0.0343 (6)	−0.0156 (5)	0.0079 (5)	−0.0150 (5)
O1	0.0319 (5)	0.0426 (5)	0.0525 (6)	−0.0097 (4)	0.0006 (5)	−0.0188 (5)
O2	0.0417 (6)	0.0613 (7)	0.0626 (8)	−0.0254 (5)	0.0011 (5)	−0.0304 (6)
O3	0.0546 (7)	0.0542 (6)	0.0554 (7)	−0.0307 (6)	0.0112 (6)	−0.0284 (6)

### Geometric parameters (Å, º)

**Table:**

C1—C2	1.383 (2)	C20—H20A	0.9700
C1—C6	1.385 (2)	C20—H20B	0.9700
C1—H1	0.9300	C21—N1	1.4645 (18)
C2—C3	1.369 (3)	C21—C22	1.518 (2)
C2—H2	0.9300	C21—C29	1.5548 (19)
C3—C4	1.377 (3)	C21—C30	1.5998 (18)
C3—H3	0.9300	C22—C23	1.380 (2)
C4—C5	1.376 (3)	C22—C27	1.395 (2)
C4—H4	0.9300	C23—C24	1.387 (2)
C5—C6	1.385 (2)	C23—H23	0.9300
C5—H5	0.9300	C24—C25	1.373 (2)
C6—C7	1.475 (2)	C24—H24	0.9300
C7—N5	1.3354 (18)	C25—C26	1.379 (3)
C7—C15	1.4146 (19)	C25—H25	0.9300
C8—C9	1.369 (2)	C26—C27	1.372 (2)
C8—C13	1.380 (2)	C26—H26	0.9300
C8—N4	1.4137 (18)	C28—N3	1.446 (2)
C9—C10	1.385 (2)	C28—H27A	0.9600
C9—H9	0.9300	C28—H27B	0.9600
C10—C11	1.364 (3)	C28—H27C	0.9600
C10—H10	0.9300	C29—O3	1.2157 (18)
C11—C12	1.356 (3)	C29—N3	1.3553 (19)
C11—H11	0.9300	C30—C38	1.5204 (19)
C12—C13	1.380 (2)	C30—C31	1.5305 (18)
C12—H12	0.9300	C31—O2	1.2136 (17)
C13—H13	0.9300	C31—C32	1.476 (2)
C14—N4	1.3465 (18)	C32—C37	1.387 (2)
C14—C15	1.365 (2)	C32—C33	1.397 (2)
C14—H14	0.9300	C33—C34	1.376 (3)
C15—C16	1.4952 (19)	C33—H32	0.9300
C16—C17	1.5519 (19)	C34—C35	1.380 (3)
C16—C30	1.5678 (18)	C34—H33	0.9300
C16—H16	0.9800	C35—C36	1.374 (3)
C17—N1	1.4587 (19)	C35—H34	0.9300
C17—C18	1.519 (2)	C36—C37	1.387 (2)
C17—H17	0.9800	C36—H35	0.9300
C18—C19	1.502 (3)	C37—O1	1.3595 (17)
C18—H18A	0.9700	C38—O1	1.4311 (17)
C18—H18B	0.9700	C38—H37A	0.9700
C19—C20	1.486 (3)	C38—H37B	0.9700
C19—H19A	0.9700	C27—N3	1.400 (2)
C19—H19B	0.9700	N4—N5	1.3582 (16)
C20—N1	1.476 (2)		
			
C2—C1—C6	120.59 (17)	N1—C21—C29	103.59 (11)
C2—C1—H1	119.7	C22—C21—C29	101.45 (11)
C6—C1—H1	119.7	N1—C21—C30	106.50 (11)
C3—C2—C1	120.08 (18)	C22—C21—C30	121.01 (11)
C3—C2—H2	120.0	C29—C21—C30	110.50 (11)
C1—C2—H2	120.0	C23—C22—C27	118.57 (13)
C2—C3—C4	119.79 (18)	C23—C22—C21	133.02 (13)
C2—C3—H3	120.1	C27—C22—C21	108.19 (12)
C4—C3—H3	120.1	C22—C23—C24	119.54 (14)
C5—C4—C3	120.40 (19)	C22—C23—H23	120.2
C5—C4—H4	119.8	C24—C23—H23	120.2
C3—C4—H4	119.8	C25—C24—C23	120.49 (16)
C4—C5—C6	120.44 (18)	C25—C24—H24	119.8
C4—C5—H5	119.8	C23—C24—H24	119.8
C6—C5—H5	119.8	C24—C25—C26	121.14 (15)
C5—C6—C1	118.64 (14)	C24—C25—H25	119.4
C5—C6—C7	119.68 (14)	C26—C25—H25	119.4
C1—C6—C7	121.67 (14)	C27—C26—C25	117.91 (15)
N5—C7—C15	111.59 (12)	C27—C26—H26	121.0
N5—C7—C6	119.80 (13)	C25—C26—H26	121.0
C15—C7—C6	128.29 (13)	N3—C28—H27A	109.5
C9—C8—C13	119.92 (15)	N3—C28—H27B	109.5
C9—C8—N4	120.33 (14)	H27A—C28—H27B	109.5
C13—C8—N4	119.74 (14)	N3—C28—H27C	109.5
C8—C9—C10	119.34 (18)	H27A—C28—H27C	109.5
C8—C9—H9	120.3	H27B—C28—H27C	109.5
C10—C9—H9	120.3	O3—C29—N3	124.73 (14)
C11—C10—C9	120.95 (19)	O3—C29—C21	126.96 (13)
C11—C10—H10	119.5	N3—C29—C21	108.23 (12)
C9—C10—H10	119.5	C38—C30—C31	106.51 (11)
C12—C11—C10	119.30 (17)	C38—C30—C16	112.90 (11)
C12—C11—H11	120.4	C31—C30—C16	110.79 (11)
C10—C11—H11	120.4	C38—C30—C21	115.00 (11)
C11—C12—C13	121.15 (18)	C31—C30—C21	108.62 (10)
C11—C12—H12	119.4	C16—C30—C21	103.01 (10)
C13—C12—H12	119.4	O2—C31—C32	121.27 (13)
C12—C13—C8	119.34 (17)	O2—C31—C30	122.10 (13)
C12—C13—H13	120.3	C32—C31—C30	116.62 (12)
C8—C13—H13	120.3	C37—C32—C33	118.30 (15)
N4—C14—C15	108.14 (13)	C37—C32—C31	120.90 (13)
N4—C14—H14	125.9	C33—C32—C31	120.78 (15)
C15—C14—H14	125.9	C34—C33—C32	120.66 (19)
C14—C15—C7	104.05 (12)	C34—C33—H32	119.7
C14—C15—C16	126.24 (13)	C32—C33—H32	119.7
C7—C15—C16	129.14 (13)	C33—C34—C35	119.85 (17)
C15—C16—C17	110.44 (11)	C33—C34—H33	120.1
C15—C16—C30	117.73 (11)	C35—C34—H33	120.1
C17—C16—C30	105.55 (10)	C36—C35—C34	120.85 (18)
C15—C16—H16	107.6	C36—C35—H34	119.6
C17—C16—H16	107.6	C34—C35—H34	119.6
C30—C16—H16	107.6	C35—C36—C37	119.11 (19)
N1—C17—C18	104.84 (12)	C35—C36—H35	120.4
N1—C17—C16	106.34 (11)	C37—C36—H35	120.4
C18—C17—C16	114.24 (13)	O1—C37—C32	121.25 (13)
N1—C17—H17	110.4	O1—C37—C36	117.52 (15)
C18—C17—H17	110.4	C32—C37—C36	121.23 (15)
C16—C17—H17	110.4	O1—C38—C30	113.00 (11)
C19—C18—C17	103.82 (13)	O1—C38—H37A	109.0
C19—C18—H18A	111.0	C30—C38—H37A	109.0
C17—C18—H18A	111.0	O1—C38—H37B	109.0
C19—C18—H18B	111.0	C30—C38—H37B	109.0
C17—C18—H18B	111.0	H37A—C38—H37B	107.8
H18A—C18—H18B	109.0	C17—N1—C21	106.82 (11)
C20—C19—C18	106.76 (15)	C17—N1—C20	108.47 (12)
C20—C19—H19A	110.4	C21—N1—C20	120.70 (12)
C18—C19—H19A	110.4	C26—C27—C22	122.34 (15)
C20—C19—H19B	110.4	C26—C27—N3	127.43 (14)
C18—C19—H19B	110.4	C22—C27—N3	110.23 (13)
H19A—C19—H19B	108.6	C29—N3—C27	111.44 (12)
N1—C20—C19	106.76 (14)	C29—N3—C28	123.50 (14)
N1—C20—H20A	110.4	C27—N3—C28	125.06 (14)
C19—C20—H20A	110.4	C14—N4—N5	111.74 (11)
N1—C20—H20B	110.4	C14—N4—C8	126.92 (12)
C19—C20—H20B	110.4	N5—N4—C8	121.34 (12)
H20A—C20—H20B	108.6	C7—N5—N4	104.48 (11)
N1—C21—C22	112.43 (11)	C37—O1—C38	112.91 (11)
			
C6—C1—C2—C3	0.0 (3)	N1—C21—C30—C16	16.80 (13)
C1—C2—C3—C4	1.8 (3)	C22—C21—C30—C16	146.76 (12)
C2—C3—C4—C5	−1.9 (4)	C29—C21—C30—C16	−95.07 (12)
C3—C4—C5—C6	0.2 (3)	C38—C30—C31—O2	−153.47 (14)
C4—C5—C6—C1	1.6 (3)	C16—C30—C31—O2	−30.33 (18)
C4—C5—C6—C7	−177.19 (17)	C21—C30—C31—O2	82.13 (16)
C2—C1—C6—C5	−1.7 (2)	C38—C30—C31—C32	27.90 (16)
C2—C1—C6—C7	177.10 (15)	C16—C30—C31—C32	151.04 (12)
C5—C6—C7—N5	−37.9 (2)	C21—C30—C31—C32	−96.50 (13)
C1—C6—C7—N5	143.31 (14)	O2—C31—C32—C37	−179.59 (14)
C5—C6—C7—C15	134.98 (17)	C30—C31—C32—C37	−0.94 (19)
C1—C6—C7—C15	−43.8 (2)	O2—C31—C32—C33	−1.3 (2)
C13—C8—C9—C10	0.1 (3)	C30—C31—C32—C33	177.34 (13)
N4—C8—C9—C10	−179.90 (16)	C37—C32—C33—C34	−0.6 (2)
C8—C9—C10—C11	−0.4 (3)	C31—C32—C33—C34	−178.92 (16)
C9—C10—C11—C12	0.8 (3)	C32—C33—C34—C35	0.2 (3)
C10—C11—C12—C13	−0.8 (3)	C33—C34—C35—C36	0.3 (3)
C11—C12—C13—C8	0.5 (3)	C34—C35—C36—C37	−0.3 (3)
C9—C8—C13—C12	−0.2 (2)	C33—C32—C37—O1	−178.60 (13)
N4—C8—C13—C12	179.84 (15)	C31—C32—C37—O1	−0.3 (2)
N4—C14—C15—C7	−1.22 (15)	C33—C32—C37—C36	0.6 (2)
N4—C14—C15—C16	−173.21 (13)	C31—C32—C37—C36	178.93 (14)
N5—C7—C15—C14	1.20 (16)	C35—C36—C37—O1	179.05 (16)
C6—C7—C15—C14	−172.17 (14)	C35—C36—C37—C32	−0.2 (3)
N5—C7—C15—C16	172.87 (13)	C31—C30—C38—O1	−57.50 (14)
C6—C7—C15—C16	−0.5 (2)	C16—C30—C38—O1	−179.31 (10)
C14—C15—C16—C17	57.39 (18)	C21—C30—C38—O1	62.87 (15)
C7—C15—C16—C17	−112.57 (15)	C18—C17—N1—C21	156.51 (12)
C14—C15—C16—C30	−63.88 (18)	C16—C17—N1—C21	35.16 (14)
C7—C15—C16—C30	126.16 (15)	C18—C17—N1—C20	24.96 (16)
C15—C16—C17—N1	−151.63 (12)	C16—C17—N1—C20	−96.39 (14)
C30—C16—C17—N1	−23.37 (14)	C22—C21—N1—C17	−167.23 (11)
C15—C16—C17—C18	93.24 (15)	C29—C21—N1—C17	84.06 (13)
C30—C16—C17—C18	−138.51 (13)	C30—C21—N1—C17	−32.52 (13)
N1—C17—C18—C19	−30.83 (17)	C22—C21—N1—C20	−42.87 (17)
C16—C17—C18—C19	85.18 (17)	C29—C21—N1—C20	−151.58 (13)
C17—C18—C19—C20	25.5 (2)	C30—C21—N1—C20	91.84 (14)
C18—C19—C20—N1	−10.8 (2)	C19—C20—N1—C17	−9.05 (19)
N1—C21—C22—C23	71.02 (19)	C19—C20—N1—C21	−132.63 (16)
C29—C21—C22—C23	−178.92 (15)	C25—C26—C27—C22	0.5 (2)
C30—C21—C22—C23	−56.3 (2)	C25—C26—C27—N3	−179.74 (15)
N1—C21—C22—C27	−103.33 (14)	C23—C22—C27—C26	−1.3 (2)
C29—C21—C22—C27	6.73 (14)	C21—C22—C27—C26	174.04 (14)
C30—C21—C22—C27	129.33 (13)	C23—C22—C27—N3	178.97 (13)
C27—C22—C23—C24	0.9 (2)	C21—C22—C27—N3	−5.73 (16)
C21—C22—C23—C24	−172.97 (15)	O3—C29—N3—C27	179.58 (14)
C22—C23—C24—C25	0.1 (2)	C21—C29—N3—C27	2.63 (16)
C23—C24—C25—C26	−0.8 (3)	O3—C29—N3—C28	0.7 (3)
C24—C25—C26—C27	0.5 (3)	C21—C29—N3—C28	−176.27 (15)
N1—C21—C29—O3	−65.80 (18)	C26—C27—N3—C29	−177.82 (16)
C22—C21—C29—O3	177.48 (15)	C22—C27—N3—C29	1.94 (18)
C30—C21—C29—O3	47.92 (19)	C26—C27—N3—C28	1.1 (3)
N1—C21—C29—N3	111.05 (13)	C22—C27—N3—C28	−179.19 (16)
C22—C21—C29—N3	−5.66 (14)	C15—C14—N4—N5	0.90 (16)
C30—C21—C29—N3	−135.22 (12)	C15—C14—N4—C8	−179.04 (13)
C15—C16—C30—C38	2.92 (16)	C9—C8—N4—C14	167.31 (15)
C17—C16—C30—C38	−120.84 (12)	C13—C8—N4—C14	−12.7 (2)
C15—C16—C30—C31	−116.44 (13)	C9—C8—N4—N5	−12.6 (2)
C17—C16—C30—C31	119.80 (12)	C13—C8—N4—N5	167.32 (13)
C15—C16—C30—C21	127.57 (12)	C15—C7—N5—N4	−0.67 (15)
C17—C16—C30—C21	3.80 (13)	C6—C7—N5—N4	173.33 (12)
N1—C21—C30—C38	140.06 (11)	C14—N4—N5—C7	−0.13 (15)
C22—C21—C30—C38	−89.97 (15)	C8—N4—N5—C7	179.82 (12)
C29—C21—C30—C38	28.19 (16)	C32—C37—O1—C38	−28.68 (19)
N1—C21—C30—C31	−100.74 (12)	C36—C37—O1—C38	152.08 (14)
C22—C21—C30—C31	29.23 (16)	C30—C38—O1—C37	59.83 (15)
C29—C21—C30—C31	147.40 (11)		

### Hydrogen-bond geometry (Å, º)

**Table:**

*D*—H···*A*	*D*—H	H···*A*	*D*···*A*	*D*—H···*A*
C34—H33···O3^i^	0.93	2.59	3.523 (3)	178

Symmetry code: (i) *x*, *y*+1, *z*.
